# Role of carriers in the transmission of pneumonia in bighorn sheep (*Ovis canadensis*)

**DOI:** 10.1242/bio.018234

**Published:** 2016-05-16

**Authors:** Bindu Raghavan, Kayla Erickson, Abirami Kugadas, Sai A. Batra, Douglas R. Call, Margaret A. Davis, William J. Foreyt, Subramaniam Srikumaran

**Affiliations:** 1 Department of Veterinary Microbiology & Pathology, Washington State University, Pullman, WA 99163, USA; 2 Paul G. Allen School for Global Animal Health, Washington State University, Pullman, WA 99163, USA

**Keywords:** Bighorn sheep, Pneumonia, Carrier, Antibodies, *Pasteurellaceae*, *Mycoplasma*

## Abstract

In the absence of livestock contact, recurring lamb mortality in bighorn sheep (*Ovis canadensis*) populations previously exposed to pneumonia indicates the likely presence of carriers of pneumonia-causing pathogens, and possibly inadequate maternally derived immunity. To investigate this problem we commingled naïve, pregnant ewes (*n*=3) with previously exposed rams (*n*=2). Post-commingling, all ewes and lambs born to them acquired pneumonia-causing pathogens (leukotoxin-producing *Pasteurellaceae* and *Mycoplasma ovipneumoniae*), with subsequent lamb mortality between 4-9 weeks of age. Infected ewes became carriers for two subsequent years and lambs born to them succumbed to pneumonia. In another experiment, we attempted to suppress the carriage of leukotoxin-producing *Pasteurellaceae* by administering an antibiotic to carrier ewes, and evaluated lamb survival. Lambs born to both treatment and control ewes (*n*=4 each) acquired pneumonia and died. Antibody titers against leukotoxin-producing *Pasteurellaceae* in all eight ewes were ‘protective’ (>1:800 and no apparent respiratory disease); however their lambs were either born with comparatively low titers, or with high (but non-protective) titers that declined rapidly within 2-8 weeks of age, rendering them susceptible to fatal disease. Thus, exposure to pneumonia-causing pathogens from carrier ewes, and inadequate titers of maternally derived protective antibodies, are likely to render bighorn lambs susceptible to fatal pneumonia.

## Abstract (precis)

**Summary:** Previously exposed bighorn sheep can become carriers and transmit respiratory pathogens to naïve animals. Successive crops of lambs acquire pathogens and inadequate passive immunity and subsequently succumb to pneumonia.

## INTRODUCTION

Over the last several decades, pneumonia of bighorn sheep (BHS) has resulted in up to 75-90% mortality in herds across North America (Rush, 1927; Buechner, 1960; Schwantje, 1986; Festa-Bianchet, 1988; Valdez and Krausman, 1999; Miller, 2001). The main pathogens involved are *Mycoplasma ovipneumoniae* (Besser et al., 2008, 2012, 2013; Dassanayake et al., 2010) and leukotoxin-producing members of *Pasteurellaceae* (lkt+ *Pasteurellaceae*), especially *Mannheimia haemolytica* and *Bibersteinia trehalosi* (Onderka and Wishart, 1984; Miller et al., 1991; Foreyt et al., 1994; Miller, 2001; Rudolph et al., 2007). In several studies, lkt+ *Pasteurellaceae* caused fatal pneumonia in >90% affected BHS (Silflow et al., 1993; Dassanayake et al., 2009, 2013; Tomassini et al., 2009; Subramaniam et al., 2011; Shanthalingam et al., 2014). Based on the observation that leukotoxin (lkt) deletion mutants do not cause mortality in BHS (Dassanayake et al., 2009), leukotxoin produced by members of Pasteurellaceae is recognized as the most important virulence factor in BHS pneumonia. Infection with *M. ovipneumoniae* predisposes and/or precipitates fatal infection by lkt+ *Pasteurellaceae* (Besser et al., 2008, 2012, 2013; Dassanayake et al., 2010). BHS populations that are afflicted with pneumonia may have originally acquired lkt+ *Pasteurellaceae* as well as *M. ovipneumoniae* from livestock, particularly domestic sheep (Schillenger, 1937; Buechner, 1960; Coggins, 1988; Miller, 2001; George et al., 2008; Wolfe et al., 2010).

In most BHS herds, epizootic pneumonia characterized by initial all-age die-offs, transitions into an enzootic disease with recurrent lamb deaths in subsequent years (Ryder et al., 1992; Hobbs and Miller, 1992; Cassirer et al., 1996; Miller, 2001; Cassirer and Sinclair, 2007). In herds with enzootic disease, lamb recruitment is reduced drastically, sometimes for more than a decade (Sandoval et al., 1987; Foreyt, 1990; Miller et al., 1991; Singer et al., 2000; Cassirer et al., 2013). Sexual segregation of BHS during lambing (Festa-Bianchet, 1991; Ruckstuhl, 1998) suggests that disease transmission in lambs most likely occurs through contact with ewes, other lambs, and/or juveniles in the herd (Festa-Bianchet, 1991; Ruckstuhl, 1998); therefore ewes that survive epizootic pneumonia may act as carriers and serve as subsequent sources of the pneumonia-causing pathogens for susceptible lambs.

Most healthy BHS typically do not harbor lkt+ *Pasteurellaceae* in their nasopharynx but can harbor non-leukotoxin producing strains of *B. trehalosi* (Sweeney et al., 1994; Shanthalingam et al., 2014). *M. haemolytica* surface antigens are similar to those of *B. trehalosi*; therefore antibodies against the latter can cross protect against *M. hemolytica*. Healthy bighorn (BH) ewes, however, have relatively low titers of lkt-neutralizing antibodies (lkt-nAb) in their serum and colostrum compared to healthy domestic ewes (Miller et al., 1997; Kraabel et al., 1998; Herndon et al., 2011; Subramaniam et al., 2011). Consequently, BH lambs born to healthy ewes acquire lower titers of these antibodies from their dams (Miller et al., 1997; Cassirer et al., 2001; Herndon et al., 2011; Subramaniam et al., 2011). This could also be the case for antibodies against *M. ovipneumoniae* in BHS.

Carrier ewes [previously exposed to pneumonia and that continue to test PCR-positive for lkt+ *Pasteurellaceae* and/or *M. ovipneumoniae* at two consecutive samplings (two weeks apart) after the end of the initial infection or exposure period] may experience waning immunity that might translate into reduced passive immunity in lambs born to them. Most BH lambs succumb to pneumonia between 6-8 weeks of age when passive immunity has waned and adaptive immunity has not yet developed (Miller et al., 1997; Herndon et al., 2011). It is possible that a longer duration of passive immunity, achieved through higher levels of maternally derived antibodies, might protect the lambs from fatal infection during this susceptible period.

Studies on BHS pneumonia have investigated the relationship between carrier ewes and survival of their lambs with respect to either one or the other of the typical pneumonia-causing pathogens. While some studies have investigated the role of co-infection by lkt+ *Pasteurellaceae* and *M. ovipneumoniae* in adult BHS (Besser et al., 2008, 2012, 2013; Dassanayake et al., 2010), its role in lamb pneumonia is still unclear. It is also unclear whether it is carriage of pathogen by the dams, inadequate maternally derived antibody titers, rapid decline of maternally derived antibody titers, or a combination of one or more of these factors that contributes most to recurrent fatal pneumonia in BH lambs. The overall aim of this study was to determine the role of carrier animals in the development of fatal pneumonia of BH lambs. The specific objectives were to: (1) determine whether animals from pneumonia-affected herds (presumed ‘carriers’) can transmit pneumonia pathogens to other naïve animals, especially lambs, when commingled; (2) determine whether recovered animals, especially ewes, become carriers of the disease and fatally infect successive crops of lambs; and (3) determine whether elimination or suppression of carriage with respect to the ‘fatal’ pathogen (lkt+ *Pasteurellaceae*) by carrier ewes would lead to lamb survival.

## RESULTS

### Study 1: commingling of naïve, pregnant bighorn ewes with previously exposed rams

#### Pre-commingling microbial, serological and disease status of all BHS

**Fig. 1.**
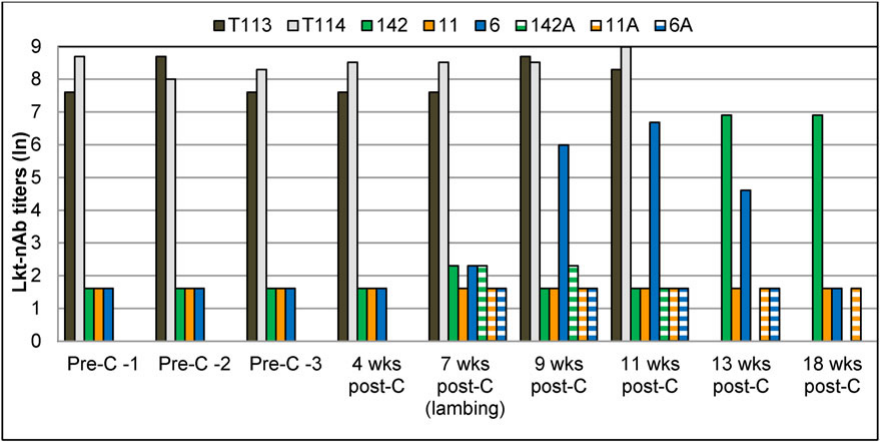
**Titers of leukotoxin-neutralizing antibodies (lkt-nAb) in bighorn carrier rams, commingled naïve pregnant ewes and lambs born to the ewes (Study 1).** Bighorn rams (*n*=2; T113 and T114) from a pneumonia-affected herd in Nevada were commingled with naïve, healthy, pregnant ewes (*n*=3; 142, 11, 6) from the WSU breeding herd. Lambs (*n*=3; 142A, 11A, 6A) were born one month post-commingling. Serum samples were collected at bi-weekly intervals. Lkt-nAb titers were measured using an MTT dye-reduction cytotoxicity inhibition assay and transformed to natural log (ln) for analysis. Each sample was tested in three independent experiments. Titers differed significantly between carrier rams and naïve ewes (*P*=0.00) based on two-sample, two-tailed *t*-test with alpha=0.05. Based on previous studies, protective titers were defined as titers >6.68 (>1:800). Pre-C, pre- commingling; Post-C, post-commingling.

**Fig. 2.**
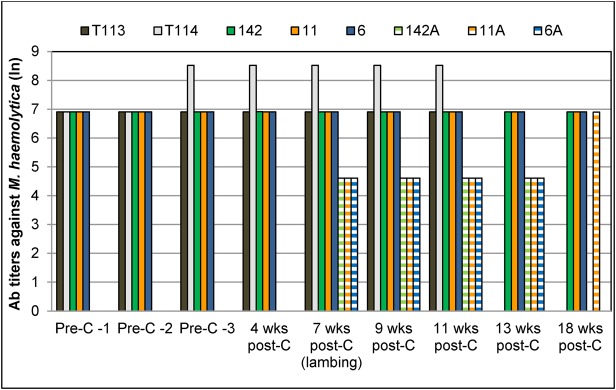
**Titers of antibodies against *M. haemolytica* surface antigens in bighorn carrier rams, commingled naïve pregnant ewes and lambs born to the ewes (Study 1).** Bighorn rams (*n*=2; T113 and T114) from a pneumonia-affected herd in Nevada were commingled with naïve, healthy, pregnant ewes (*n*=3; 11, 142, 6) from the WSU breeding herd. Lambs (*n*=3; 142A, 11A, 6A) were born one month post-commingling. Serum samples were collected bi-weekly. Antibody (Ab) titers were measured using an indirect ELISA and transformed to natural log (ln) for analysis. Each sample was tested in three independent experiments. Titers differed significantly between carrier rams and naïve ewes (*P*=0.00) based on two-sample, two-tailed *t*-test with alpha=0.05. *M. haemolytica* surface antigens are similar to those of *B. trehalosi*. Therefore, antibody titers may not necessarily reflect titers against *M. hemolytica* alone and results must be treated with caution. Pre-C, pre- commingling; Post-C, post-commingling.

Prior to commingling with carrier rams, the three ewes in the treatment group (6, 11 and 142) tested PCR-negative for lkt+ *Pasteurellaceae* and *M. ovipneumoniae* (Table 1). They had negligible titers of lkt-nAb (Fig. 1) and were seronegative for *M. ovipneumoniae* (Table 1). They also had low titers of antibodies against *M. haemolytica* surface antigens (6.9; Fig. 2). None of the ewes had any clinical signs of pneumonia or other respiratory disorders.

**Table 1.**
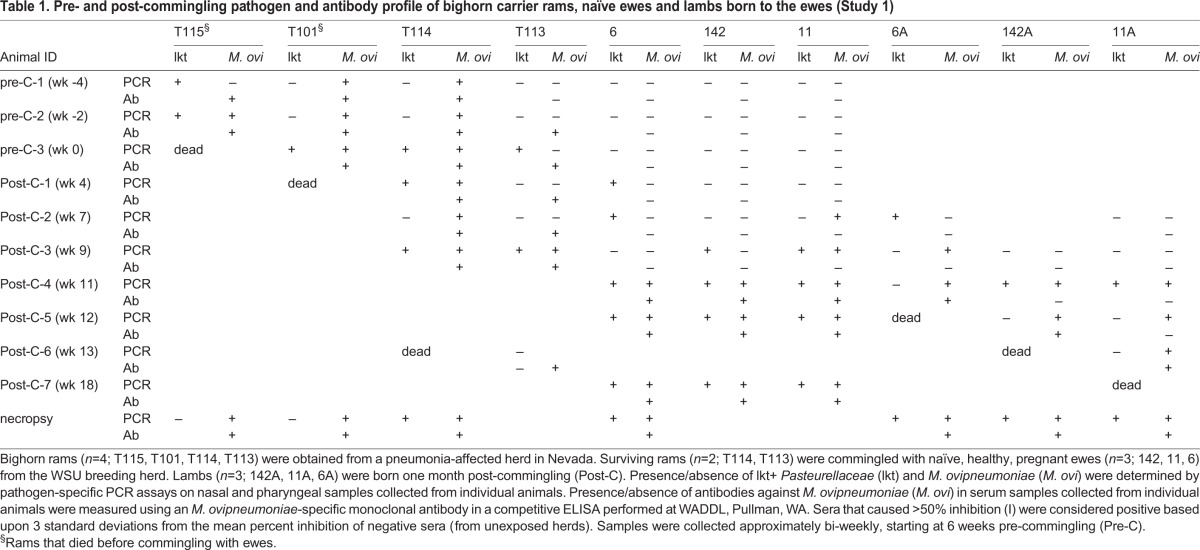
**Pre- and post-commingling pathogen and antibody profile of bighorn carrier rams, naïve ewes and lambs born to the ewes (Study 1)**

The two carrier rams (T114 and T113) used for commingling were PCR-positive for lkt+ *Pasteurellaceae* (Table 1). Both rams had very high titers of lkt-nAb throughout the study period (Fig. 1), and relatively low antibody titers against *M. haemolytica* surface antigens (6.9) prior to commingling (Fig. 2). Only ram T114 tested PCR- and sero- positive for *M. ovipneumoniae* (Table 1) prior to commingling. Ram T113 tested PCR-negative for *M. ovipneumoniae* pre-commingling but was positive post-commingling (Table 1). Both rams tested seropositive for *M. ovipneumoniae* throughout the rest of the study period (Table 1).

#### Post-commingling microbial, serological and disease status of commingled BHS

All three pregnant ewes tested PCR-positive for lkt+ *Pasteurellaceae* at 4 (Ewe 6) and 9 weeks (Ewes 11 and 142), and for *M. ovipneumoniae* at 7 (Ewe 11) and 9 weeks (Ewes 6 and 142) after commingling with the carrier rams (Table 1). The ewes continued to test PCR-positive for lkt-producing *Pasteurellaceae* and *M. ovipneumoniae* until the end of the study at 18 weeks post-commingling. All ewes showed signs of upper respiratory tract infection (mild to moderate muco-purulent nasal discharge, sneezing and coughing) soon after their lambs were born, about 7 weeks post-commingling. Ewe 6 died around 20 weeks post-commingling due to bronchopneumonia exacerbated by fractured ribs that had punctured the lungs. The other two ewes recovered from the disease but continued to test positive for the pathogens until the end of this study, at 18 weeks (5 months) post-commingling.

Lambs 6A and 11A were born to ewes 6 and 11 respectively, around 7 weeks post-commingling. Lamb 142A was born to ewe 142 approximately 9 weeks post-commingling. Lamb 6A tested PCR-positive for lkt+ *Pasteurellaceae* at birth and for *M. ovipneumoniae* at 2 weeks of age (Table 1). Lambs 11A and 142A were born naïve for both pathogens but tested positive by 4 and 2 weeks of age, respectively (Table 1). All three lambs developed signs of respiratory disease and had to be euthanized at 4, 6 and 9 weeks of age (6A, 142A and 11A, respectively). Necropsy and histopathological examination confirmed bacterial bronchopneumonia and fibrinous pleuritis in the lungs. Within affected regions of the lungs, alveolar spaces and bronchioles were filled with edema, fibrin, red blood cells, and dense collections of primarily neutrophils and eosinophils, with fewer macrophages. Bronchiolar, and occasionally alveolar, walls were disrupted by sloughed epithelium and hyperplasia. Gram-negative rods were also found in the lungs. Lesions were consistent with both leukotoxin- and *M. ovipneumoniae*-induced lung injury.

Concurrent with the development of pneumonia in the lambs, the two carrier rams also showed signs of upper respiratory tract infection. Ram T114 succumbed to the disease after 15 weeks of commingling. Necropsy and histopathological examination confirmed presence of chronic pleuropneumonia, which in concert with a more acute bronchopneumonia is likely to have led to the death of the animal.

##### Leukotoxin neutralizing antibodies (lkt-nAb)

Both carrier rams had high titers of lkt-nAb throughout the study (*P*<0.01; Fig. 1). Ewe 11 did not have any appreciable lkt-nAb titer throughout the study. Ewe 6 developed high lkt-nAb titers by about 9 weeks post-commingling, but these titers decreased to negligible levels by 17 weeks and remained low at the time of death (20 weeks post-commingling; data not shown). Ewe 142 developed high lkt-nAb titers at about 12 weeks post-commingling, which were sustained through the rest of the study. Overall, ewes had significantly higher lkt-nAb titers post-commingling compared with pre-commingling (*P*=0.015); however none of the ewes developed protective titers at parturition. This was reflected in the antibody titers of lambs at birth (*P*<0.01; Fig. 1).

##### Antibodies to surface antigens of *M. haemolytica*

Both rams had protective titers of antibodies to *M. haemolytica* surface antigens throughout the study, especially ram T114 (8.51; Fig. 2). Antibody titers of ewes, while not as high as those of the rams post-commingling (*P*<0.01), remained the same throughout the study (6.91). The lambs had very low titers (4.61) throughout the study (*P*<0.01), except lamb 11A, which had a higher titer at death (6.91; Fig. 2). *M. haemolytica* surface antigens are similar to those of *B. trehalosi*. Non-leukotoxin-producing strains of the latter are commonly found in BHS; therefore these titers may not reflect exposure to *M. haemolytica* alone and results should be treated with caution.

##### Antibodies against *M. ovipneumoniae*

The rams were seropositive for *M. ovipneumoniae* throughout the study (Table 1). The ewes became seropositive 9 weeks after commingling and stayed seropositive through the rest of the study (Table 1). The lambs were born seronegative but seroconverted at the time of death (Table 1).

### Study 2: carrier status of surviving ewes and status of their lambs with respect to pneumonia

The two surviving ewes (11 and 142) from study 1 remained free of apparent disease until the next crop of lambs (11B and 142B) was born in the summer of 2013. Ewe 11 had low lkt-nAb titers during study 1 (Fig. 1), a small peak between 10-13 months post-first infection, and a drop to negligible levels through the rest of study 2 (Table 2). Ewe 11 was also PCR-positive for *M. ovipneumoniae* through most of the period of study 2 (Table 2). Ewe 142 had protective titers (6.91) of lkt-nAb at the end of study 1 (Fig. 1) that dropped to negligible levels from the 10th until about the 27th month post-first infection (Table 2). Ewe 142 was also PCR- and sero-negative for lkt+ *Pasteurellaceae* and *M. ovipneumoniae* for almost a year, from about 10 and 13 months post-first infection, respectively.

**Table 2.**
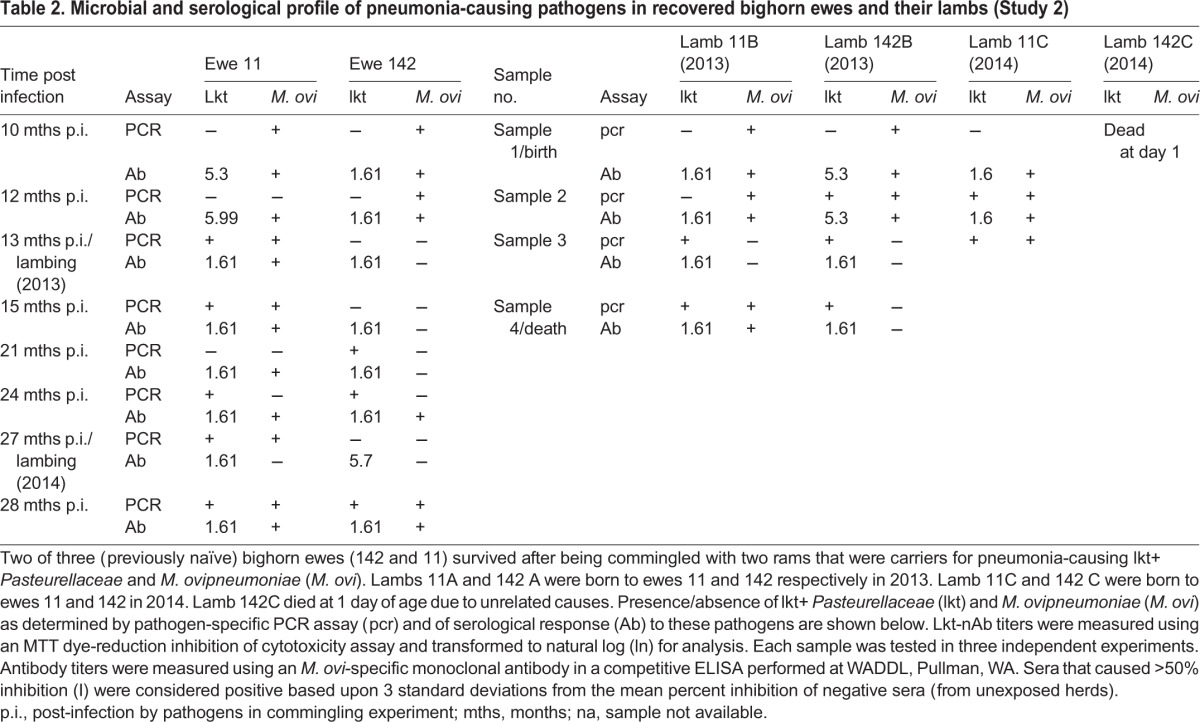
**Microbial and serological profile of pneumonia-causing pathogens in recovered bighorn ewes and their lambs (Study 2)**

Lambs 11C and 142C were born to both ewes in summer 2014; however lamb 142C died at 1 day of age due to causes unrelated to pneumonia. Lambs 11B, 142B and 11C were born naïve in terms of carriage of lkt+ *Pasteurellaceae* but acquired them by 2-4 weeks of age (Table 2). Only lamb 142B had appreciable (5.3) but not protective titers of lkt-nAb, which dropped to negligible levels by death. Lamb 11B was PCR-negative for *M. ovipneumoniae* at birth but tested positive thereafter until death (Table 2). Lambs 142B and 11C were PCR-positive for *M. ovipneumoniae* from birth until death. All lambs were born seropositive for *M. ovipneumoniae*. Lamb 11C was seropositive for *M. ovipneumoniae* from birth until death. All three lambs died due to acute bronchopneumonia within 4-10 weeks of age.

### Study 3: suppression of carriage of lkt+ *Pasteurellaceae* in enzootic pneumonia-affected Colorado bighorn ewes and impact on lamb survival

#### Pre-treatment microbial, serological and disease status of carrier ewes

**Fig. 3.**
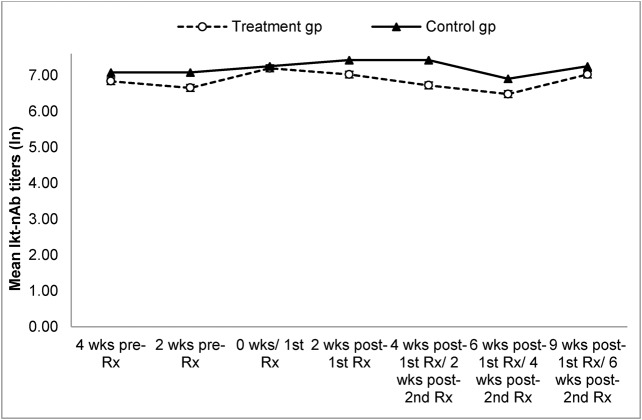
**Mean titers of neutralizing antibodies against leukotoxin (lkt-nAb) in bighorn carrier ewes in treatment and control groups (Study 3).** Eight ewes obtained from an enzootic pneumonia-affected bighorn herd in Colorado were divided into control and treatment groups (*n*=4 each) after confirming their status with respect to carriage of lkt+ *Pasteurellaceae* and *M. ovipneumoniae*. Treatment group was administered antibiotic (Zuprevo™) by sub-cutaneous injection in the neck, twice, at 28-day intervals, starting 2 weeks prior to lambing to suppress carriage of lkt+ *Pasteurellaceae*. Leukotoxin-neutralizing antibody titers were determined using MTT dye-reduction inhibition of cytotoxicity assay and transformed to natural log (ln) for analysis. Blood samples were collected at 2 weeks intervals, starting at 4 weeks prior to antibiotic treatment. Each sample was tested in three independent experiments. Each data point represents the mean titer for all samples of each group at the indicated time point. There was no significant difference between titers of control versus treatment group (*P*=0.18) or within treatment group before and after treatment (*P*=0.06). Results based on 2-way repeated measures ANOVA for former and Friedman test for latter. Error bars represent s.e.m. pre-Rx, before 1st treatment with antibiotic; 1st Rx, first treatment with antibiotic (day 0); 2nd Rx, second treatment with antibiotic (4 weeks after first treatment).

Seven of eight carrier ewes obtained from a Colorado (CO) BHS herd affected by enzootic pneumonia tested PCR-positive for lkt+ *Pasteurellaceae* (Table 3). All ewes also had high titers of lkt-nAb (6.4 to 6.91; Fig. 3). Interestingly, the control group ewes maintained high and protective titers (>6.91) of lkt-nAb throughout the study. All ewes tested PCR-positive for *M. ovipneumoniae* in at least one sampling prior to treatment (Table 3) and all were seropositive for the same (data not shown)*.* Several of the ewes had nasal discharge and a low-grade cough.

**Table 3.**
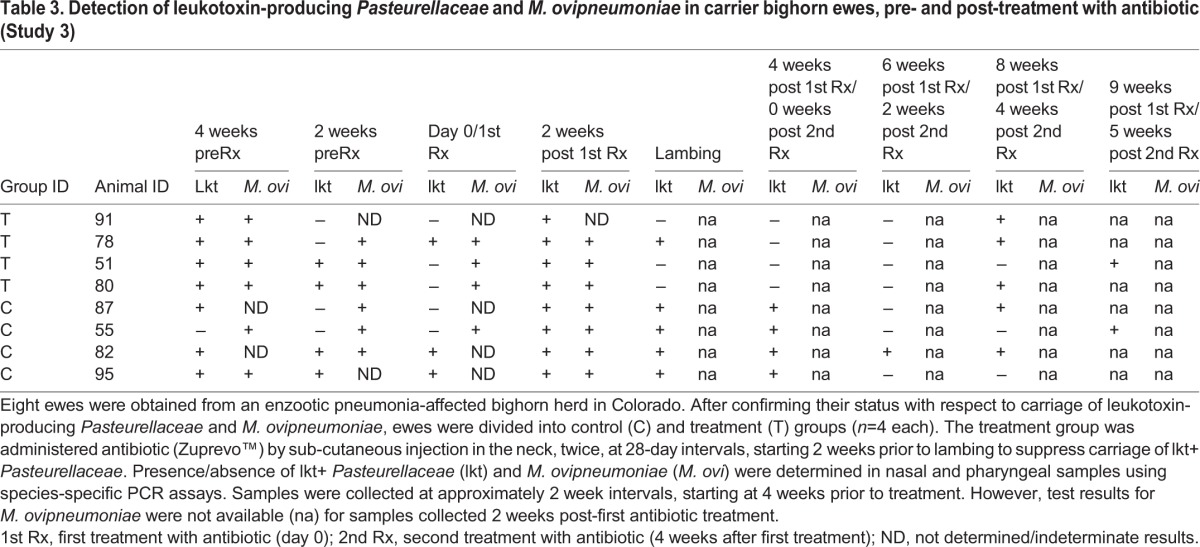
**Detection of leukotoxin-producing *Pasteurellaceae* and *M. ovipneumoniae* in carrier bighorn ewes, pre- and post-treatment with antibiotic (Study 3)**

#### Post-treatment microbial, serological and disease status of carrier ewes

Most of the treatment group ewes, except ewe 78, had no detectable lkt+ *Pasteurellacea*e between week 3 (week 4 for ewe 78) to week 6 after first administration of antibiotic (Table 3). However, lkt+ *Pasteurellacea*e were detected after week 6, (2 weeks post-second antibiotic administration) in all ewes except 51 which tested PCR-positive at 8 weeks post-first antibiotic administration (4 weeks post-second antibiotic administration). Although control group ewes did occasionally test PCR-negative for lkt+ *Pasteurellacea*e at two consecutive samplings, there was no apparent pattern (as in the treatment ewes) and they were positive during most of the study. Both treatment and control group ewes were PCR-positive for *M. ovipneumoniae* at least until lambing, after which data was not available for the same (Table 3).

Lambs were born to all ewes between 3and 4 weeks post-antibiotic treatment. However, one of the ewes (78) in the treatment group had a stillborn lamb (78A), while one of the lambs (55A) born to a control group ewe (55) died at one day of age. Of the remaining six lambs, only three tested PCR-positive for lkt+ *Pasteurellaceae*. Lamb 51A was positive for lkt+ *Pasteurellaceae* at necropsy. Only five of eight lambs tested PCR-positive for *M. ovipneumoniae* in at least one sampling, or at necropsy (Table 4).

**Table 4.**
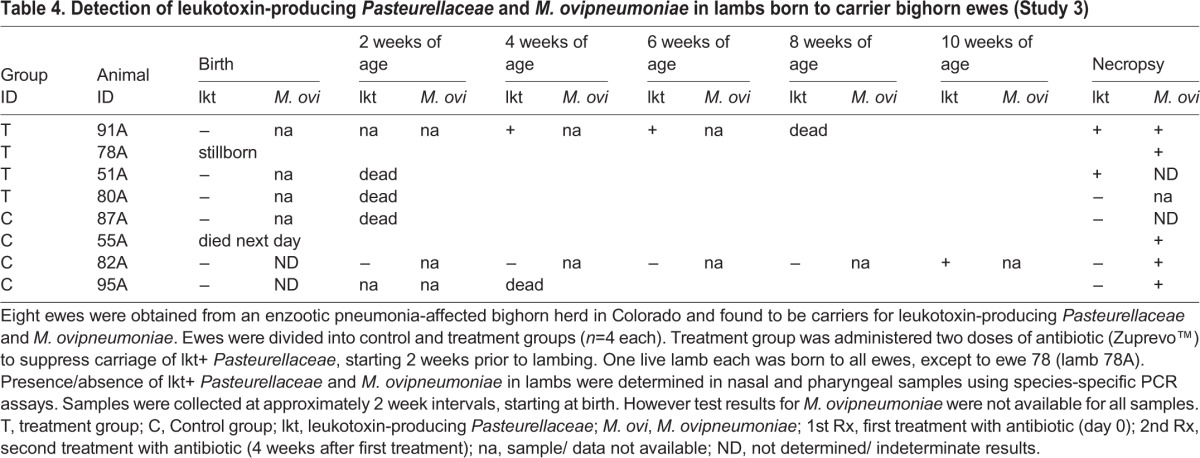
**Detection of leukotoxin-producing *Pasteurellaceae* and *M. ovipneumoniae* in lambs born to carrier bighorn ewes (Study 3)**

The treatment group ewes developed moderately high titers of lkt-nAb (Fig. 3) but there was no significant change pre- and post- treatment (*P*=0.06). There was also no significant difference between lkt-nAb titers of treatment and control ewes (*P*=0.18). All ewes, except ewe 55, were seropositive for *M. ovipneumoniae* throughout the study (data not shown). Ewe 55 was seronegative for *M. ovipneumoniae* throughout the study. All lambs were born seropositive for *M. ovipneumoniae* and died seropositive, except lamb 51A which became seronegative at 2 weeks of age (data not shown). Of the six surviving lambs, only lamb 95A (control group) was born with protective titers of lkt-nAb, while lamb 87A was born with unappreciable titers (6.68; data not shown). All other lambs were born with high (5.99) but not protective titers which declined to negligible levels by 2-3 weeks of age, and most succumbed to pneumonia at this age (data not shown). Only one control lamb (82A) survived until 10 weeks of age before dying of pneumonia.

## DISCUSSION

The initial introduction of pneumonia into BHS populations across their distribution is attributed to pathogen transmission due to close proximity with livestock, particularly domestic sheep (Rush, 1927; Schillenger, 1937; Coggins, 1988; Sandoval, 1988; Rudolph et al., 2007; George et al., 2008). Nevertheless, several BHS herds continue to exhibit enzootic disease even when no domestic sheep or livestock are present in the vicinity (Ryder et al., 1992; Hobbs and Miller, 1992; Cassirer et al., 1996; Miller, 2001; Cassirer and Sinclair, 2007). This suggests that BHS that survive an epizootic become carriers and transmit disease to other animals in the herd. They can also act as sources of infection to other herds, especially through dispersing males (Cassirer and Sinclair, 2007); however it is not clear whether they become carriers, and/or develop high antibody titers, with respect to either one (lkt+ *Pasteurellaceae* or *M. ovipneumoniae*) or both pathogens. It is also unclear why carrier ewes are able to develop adequate antibody titers against the pathogens to overcome the disease and survive, yet their lambs succumb to the disease (Foreyt et al., 1994; Singer et al., 2000; Cassirer et al., 2001, 2013). Several studies have investigated the role of antibodies against lkt+ *M. haemolytica* in carrier ewes and lambs, (Miller et al., 1997; Kraabel et al., 1998; Ward et al., 1992; Cassirer et al., 2001) but these studies did not investigate *M. ovipneumoniae* co-infection. Other studies of *M. ovipneumoniae* in BHS pneumonia mainly involved adults (Besser et al., 2008; Dassanayake et al., 2010). The few studies involving *M. ovipneumoniae* in live lambs did not investigate lkt+ *Pasteurellaceae* as an agent of fatal pneumonia in BHS (Besser et al., 2012, 2013). Thus, there is a need to establish an experimental model that mimics the transition from epizootic pneumonia into enzootic pneumonia in BHS. Such a model would permit better understanding of the role of both lkt+ *Pasteurellaceae* and *M. ovipneumoniae*, as well as that of maternally derived antibodies in the survival of pneumonic BH lambs.

In this study, three pregnant, healthy BH ewes were commingled with two rams known to be carriers for pneumonia-causing pathogens. The ewes acquired pathogens and developed mild to severe pneumonia within 4 weeks of commingling. Two ewes developed high titers of lkt-nAb, but did not do so until after the lambs were born. Antibodies to *M. ovipneumoniae* also developed after parturition in all ewes, and lambs born to them were seronegative except at death. This suggested that the lambs did not receive adequate maternally derived antibody titers. Active immune response to leukotoxin and *M. haemolytica* seems to develop only by about 6-8 weeks of age in bighorn sheep (Miller et al., 1997). While the lambs probably had started to develop an immune response to *M. ovipneumoniae* as indicated by seropositivity at death, they were clearly unable to mount an adequate response against lkt+ *M. haemolytica*. Based on previous studies (Miller et al., 1997; Kraabel et al., 1998; Ward et al., 1992; Cassirer et al., 2001; Herndon et al., 2011; Subramaniam et al., 2011), we have defined protective titers of lkt-nAb as being >6.68 (>1:800). Higher titers of maternally derived antibodies might have protected the lambs from fatal pneumonia until they were able to develop an active immune response to the pathogens. Studies on calves and lambs have determined that the titer of maternally derived immunoglobulins declines rapidly over time and that this decline is dependent on the specific pathogen against which antibodies are produced in the dam (Sasaki et al., 1977; Jones et al., 1982; Klobasa and Werhahn, 1989; Watson, 1992; Dominguez et al., 2001). Further studies are necessary to identify the minimal titer of maternally derived antibodies needed to protect BH lambs from fatal pneumonia.

Through most of the subsequent two years, surviving ewe 11 remained PCR-positive for both lkt+ *Pasteurellaceae* and *M. ovipneumoniae*. Surviving ewe 142 was PCR-negative for both pathogens until parturition in the year following the commingling, and also had low lkt-nAb titers and undetectable titers against *M. ovipneumoniae*. The re-detection of the pathogens soon after this period indicates a waning immunity that potentially allows pathogens to rebound. Lambs born to these ewes in successive two years were seropositive for *M. ovipneumoniae* from birth until death; however they had low titers of lkt-nAb. All the lambs died due to acute bronchopneumonia within 4-10 weeks of age. This suggests a greater role for lkt+ *Pasteurellaceae* in bighorn lamb pneumonia than previously considered.

Thus, it seems that at least one of the ewes (11) from study 1 had become a carrier for the pathogens. The other (142) probably cleared initial infection but due to close proximity with a carrier and possibly, waning immunity, might have been re-exposed to the pathogens. It is possible that if ewe 142 had been housed in isolation, her lamb might have survived; however under natural conditions, lambs typically interact with other animals (‘nursery groups’; Festa-Bianchet, 1991; Ruckstuhl, 1998) apart from just their dams. Hence, group-level prevalence and shedding is probably more important than the individual carrier state in BH lamb pneumonia. It is also possible that if the lambs had been born with protective levels of lkt-nAb and maintained these at least until 10 weeks of age, they might have survived fatal pneumonia; however our sample size was too small to substantiate this theory.

Results from study 2 indicated that lkt+ *Pasteurellaceae* might play an important role in fatal pneumonia of BH lambs. Therefore we hypothesized that decline in, or elimination of, carriage of at least lkt+ *Pasteurellaceae* might lead to better lamb survival. Our third study tested this hypothesis by attempting to eliminate or suppress carriage of lkt+ *Pasteurellaceae* in pregnant, carrier bighorn ewes using an antibiotic. We used a macrolide [*Zuprevo*™; (Tildipirosin, Merck Animal Health, Madison, NJ)] that is known to maintain high, prolonged concentrations in the lungs and bronchial fluids of cattle (for up to 28 and 21 days, respectively; Menge et al., 2012). In our experiment we were only able to achieve a transient decline in carriage for about 4 weeks post-first administration and this did not occur until after the lambs were born (and thus, exposed). There was no change in carriage after the second administration of the drug. Lambs were born with apparently inadequate (non-protective) titers of lkt-nAb and the antibiotic treatment for the ewes had no impact on lamb survival against pneumonia.

Our study confirms that BHS previously exposed to pneumonia epizootics are capable of transmitting pathogens and disease to naïve animals, thus they can become carriers of pneumonia-causing pathogens. Although carrier ewes are able to mount a seemingly protective antibody response, titers of maternally derived antibodies were inadequate to protect the lambs from fatal pneumonia. Even when lambs acquire high titers of maternally derived antibodies (as seen in lambs 142B, 95A, 51A, 91A, 80A and 82A), the titers probably sharply decline soon after birth rendering the lambs susceptible to fatal disease; however additional data is required to confirm this hypothesis. These findings have serious implications for bighorn sheep management practices where animals from healthy herds, unaffected by pneumonia, are transplanted to pneumonia-affected herds to improve productivity and survival. Our study suggests that such practices result in the naïve, healthy animals becoming infected by survivors from the original herd, leading to an enzootic state with continuing BHS mortality and persistence of pneumonia pathogens.

The involvement of multiple pathogens in the etiology of BH pneumonia makes it difficult to fully understand the disease process, especially in lambs. For one of these pathogens (*M. ovipneumoniae*) currently available serological tests do not allow determination of individual animal titers, thus we are unable to determine if lambs receive adequate titers of maternally derived antibodies against *M. ovipneumoniae*, or indeed if ewes themselves have protective titers of antibodies against this pathogen. It therefore becomes difficult to ascertain whether lambs acquire adequate titers of maternally derived antibodies for one or both pathogens, and how that affects survival; however our studies suggest that lkt+ *Pasteurellaceae*, and immune response to the same, might play a more important role in BH lamb pneumonia than was thought previously. Further studies are necessary to investigate whether high maternally derived antibody titers (e.g. through use of vector-based vaccines in ewes) against one or both pathogens would be able to protect BH lambs from fatal pneumonia, even in the face of continued exposure from carrier ewes. Further studies are also required to identify the role of infection by either lkt+ *Pasteurellaceae*, or *M. ovipneumoniae*, by itself in BH lamb pneumonia.

## MATERIALS AND METHODS

### Experimental set up and design

#### Study 1

Our first hypothesis was that animals from a herd that recovered from pneumonia are presumed carriers and can transmit pneumonia pathogens to other naïve animals, especially lambs. For this study, in spring 2012 we acquired four naturally recovered wild Rocky Mountain BHS rams (T113, T114, T115 and T101), aged 2-4 years and suspected to be carriers of pneumonia pathogens, from a pneumonia-affected (2009-2010) herd in Nevada. One of the rams (T115) died during first capture after arrival at the Washington State University (WSU) facility. Three hand-reared, pregnant and healthy 4-year-old Rocky Mountain BH ewes (6, 11 and 142), previously unexposed to pneumonia pathogens (naïve), were acquired from the WSU breeding herd for commingling with the rams. The number of animals was restricted to three due to shortage of pregnant breeding ewes available for the study. A second ram (T101) died due to acute bronchopneumonia just before commingling with the ewes. The remaining two rams were then commingled with the three ewes for 16 weeks. Lambs (6A, 11A and 142A) were born to these ewes starting around 7 weeks post-commingling. All animals, including the two rams, were housed together until 9 months post-commingling. We monitored development and progress of pneumonia in the ewes and in lambs born to them.

#### Study 2

Our second hypothesis was that recovered ewes become carriers of pneumonia-causing pathogens, transmitting pathogens and fatal pneumonia to successive crops of lambs. To test this hypothesis, we isolated and monitored the two surviving ewes (11 and 142) from our first experiment at WSU. These ewes had been in contact with the surviving ram (T113) from the first study until early January 2013 and hence were pregnant when isolated. T113 was euthanized in April 2013. In 2014, the ewes were mated with another ram from our herd that was naïve for all pathogens but had been vaccinated against lkt+ *Pasteurellaceae.* We monitored the lambs (11B, 142B and 11C) born to them in two successive years (2013 and 2014), for pneumonia. Samples were collected opportunistically from the ewes and at birth and every two weeks thereafter from the lambs.

#### Study 3

Our third hypothesis was that suppression of carriage of lkt+ *Pasteurellaceae* in carrier ewes will protect BH lambs from fatal pneumonia. To test this hypothesis, we acquired eight pregnant Rocky Mountain BH ewes (approximately 6-8 years old) from a pneumonia-affected herd (Sirochman et al., 2012) in Colorado in spring 2013. The animals were housed at the Tom Thorne and Beth Williams Wildlife Research Center of the Wyoming Game and Fish Department. After screening them for lkt+ *Pasteurellaceae* and *M. ovipneumoniae*, we divided the ewes into a control and a treatment group, with four animals each. Animals were allocated to each group based on random picking of numbers, associated with each animal's ID, by a third-party unrelated to the study. The treatment group ewes (91, 78, 51, 80) were treated with an antibiotic Zuprevo™ (Tildipirosin), by subcutaneous injection at 1 ml/100 lb body weight (4 mg/kg) in the neck region (as recommended by the manufacturer) at day 0 (1st treatment; 2 weeks prior to lambing) and again at 4 weeks post-first injection (2nd treatment). No lesions were noticed at injection site and none of the ewes suffered any reactions from the drug or delivery method. Zuprevo™ (Tildipirosin) is a long-acting macrolide that is proven to be highly effective against *Pasteurellaceae* in cattle (Menge et al., 2012). It is absorbed fast and maintains prolonged concentrations in the lung and in bronchial fluids; however it is not known to be effective against *M. ovipneumoniae*. Nasal and pharyngeal swabs and blood samples were collected at 2 week intervals, starting from 4 weeks prior to antibiotic treatment, from all ewes. The same sample types were collected from lambs, born to these ewes, at birth and every 2 weeks thereafter, until death of the lambs. All animals were monitored for development of pneumonia.

### Power and sample size analysis

Considering the species used in the study, we strived to use as few animals as strictly necessary and possible. For study 1, a one-way ANOVA model, with maximum difference in antibody titers of 5.3 (ln of titers) and standard deviation of 1.6, indicated that sample size of three animals would be sufficient to obtain statistically significant results with a power of 0.95 at alpha=0.05. For study 3, a post priori one-way ANOVA model, with maximum difference in antibody titers of 1.9 (ln of titers) and standard deviation of 0.48, indicated that sample size of four animals per group would be sufficient to obtain statistically significant results with a power of 0.99 at alpha=0.05.

### Ethical statement, capture and care of animals used

All experiments were conducted under protocols approved by WSU's Institutional Animal Care and Use Committee (IACUC; ASAF# 04388-001), at WSU and at the Tom Thorne and Beth Williams Wildlife Research Center of the Wyoming Game and Fish Department WY. Animals were housed in IACUC-approved, outdoor bighorn enclosures made of wildlife-proof fencing for study 1, indoor weather-proof and temperature-controlled pens for study 2 and 3, with artificial lighting simulating natural light/dark patterns. They were fed on a diet of alfalfa hay, grass and pelleted feed specially formulated for bighorns to meet all nutritional requirements. Average weight of rams=220 lbs, ewes=140 lbs, lambs=9-40 lbs. All animals were handled by trained individuals by first running into capture nets (rams) or rounding up into squeeze pens followed by physical restraint. All animals were blindfolded and vital signs monitored for capture-related stress or trauma. All sick animals were either treated with appropriate antibiotics by attending veterinarians, or euthanized depending on severity of condition in accordance with animal care protocol.

### Collection of samples from live animals

Nasal and pharyngeal swabs were collected from all animals in Fisher's solid modified Amies transport media (Fisher Scientific, PA, USA) for *Pasteurellaceae*, as well as in *Mycoplasma* broth (Hardy Diagnostics, CA, USA) for *M. ovipneumoniae*. *Pasteurellaceae* samples were plated on brain heart infusion (BHI; Remel, Lenexa, KS) agar plates within six hours of collection and incubated at 37°C and 5% CO_2_ overnight. *Mycoplasma* broth was submitted to Washington Animal Disease Diagnostic Laboratory (WADDL), Pullman, WA, (Study 1 and 2) and to the Wyoming State Veterinary Laboratory (WSVL) Laramie, WY (Study 3) for PCR identification of *M. ovipneumoniae*. Blood was also collected at the same time from all animals through venipuncture, and serum isolated and stored at −20°C until further analysis.

### Necropsy

Animals that died or were euthanized during the experiment were necropsied by a Board-certified pathologist at WADDL, Pullman, WA or at the Colorado Parks and Wildlife Facility at Fort Collins, CO. Gross and histological examinations were conducted. Tissue and swab samples were also collected from nasal cavity, pharynx, trachea, bronchi, lungs, tympanic bullae and frontal sinuses in appropriate media for *Pasteurellaceae* and *M. ovipneumoniae*, respectively.

### Isolation and identification of *Pasteurellaceae* by polymerase chain reaction (PCR) assay

Colonies suspected to be lkt+ *Pasteurellaceae* (*M. haemolytica* or *B. trehalosi*) from each plated swab sample were further plated on a fresh BHI agar plate and incubated at 37°C and 5% CO_2_ overnight. A multiplex PCR assay to detect *Mannheimia* sp. and *B. trehalosi* was performed on bacterial colonies from each sample, using methods and primers described in Dassanayake et al. (2010). *M. haemolytica* specific PCR was carried out on *Mannheimia*-positive isolates using methods and primers described in Shanthalingam et al. (2014). Positive *M. haemolytica* and *B. trehalosi* isolates were further screened for presence of leukotoxin gene by PCR assay to amplify lktA gene using primer set-2 described in Shanthalingam et al. (2014). PCR results were visualized by electrophoresis on a 1.5% agarose gel stained with ethidium bromide.

### Isolation and identification of *M. ovipneumoniae* by PCR

Swabs collected in *Mycoplasma* broth were tested by WADDL and Wyoming Game and Fish Department Wildlife Health Laboratory, for *M. ovipneumoniae* by PCR amplification using methods and primers described in McAuliffe et al. (2003) and Besser et al. (2008).

### Detection of cytotoxicity of *lktA* gene-positive *Pasteurellaceae* isolates

Isolates positive for *lktA* gene were cultured and the culture supernatant (leukotoxin) tested for cytotoxic activity against bovine lymphoma-3 (BL-3) cells using an MTT-dye reduction cytotoxicity assay as described by Gentry and Srikumaran (1991). The percent cytotoxicity was calculated as follows:
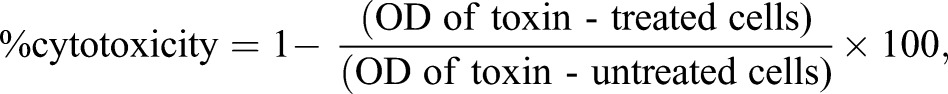
where OD=optical density measured at 540 nm.

The BL-3 cells were obtained from ATCC (hence considered authenticated and tested for contamination).

### Antibodies against surface antigens of *Pasteurellaceae*

Antibodies against *M. haemolytica* surface antigens were detected by standard indirect ELISA as described by Subramaniam et al. (2011). The OD of the final product was measured at 405 nm (OD405) using an ELISA plate reader. The highest dilution of serum yielding an OD405 value equal to or greater than twice that given by a comparable dilution of negative control serum were considered the antibody titer of the serum. In previous studies in our lab, it was determined that mean titers of antibodies against surface antigens of *M. haemolytica* (serotype A2) in unexposed, healthy BHS were 1:1400 (Herndon et al., 2011) and that in BHS vaccinated against lkt+ *Pasteurellaceae* were up to 1:4000 (Subramaniam et al., 2011). Therefore, for this study we have considered titers of antibodies against surface antigens of *M. haemolytica*>1:2000 to be protective.

### Antibodies against leukotoxin

Serum lkt-nAb were measured using an inhibition of cytotoxicity assay as described in Subramaniam et al. (2011). This assay was performed as described for the MTT dye-reduction cytotoxicity assay with the exception that lkt (at dilution that gives 50% lysis of BL-3 cells) was pre-incubated with serial dilutions of the serum samples for one hour at 4°C before adding to the BL-3 cells. Percentage inhibition of cytotoxicity by serum dilutions was calculated as follows:
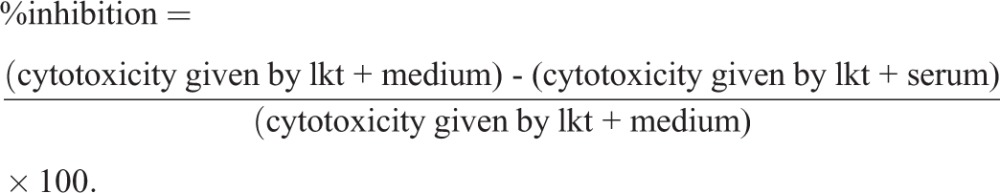
The lkt-nAb titer of a serum sample was the highest dilution that gave 50% inhibition of cytotoxicity. Based on previous studies of exposure, vaccination and/or challenge against lkt+ *M. haemolytica* in BHS (Miller et al., 1997; Kraabel et al., 1998; Ward et al., 1992; Cassirer et al., 2001; Herndon et al., 2011; Subramaniam et al., 2011), we have considered lkt-nAb titers over 1:800 to be protective and those less than 1:50 as unappreciable or negligible.

### Antibodies against *M. ovipneumoniae*

Antibodies to surface antigens of *M. ovipneumoniae* were determined using a competitive ELISA performed at WADDL, Pullman, WA. This test uses a *M. ovipneumoniae*-specific monoclonal antibody. Sera that caused >50% inhibition (I) were considered positive based upon three standard deviations from the mean percent inhibition of negative sera (from unexposed herds). Results are reported as ‘antibody not detected’ (% I<40%); ‘antibody detected at levels consistent with previous exposure or current infection with *M. ovipneumoniae*’ (% I≥50%); ‘antibody detection indeterminate to establishment of correlation with *M. ovipneumoniae* infection’ (% I=40% to 50%) (Ziegler et al., 2014).

### Statistical analysis

Antibody titer for each sample was the mean of three independent experiments. All titers were converted to natural logarithms to meet assumptions of normality of observed data. Differences in titers of lkt-nAb and antibodies to surface antigens of lkt+ *Pasteurellaceae* between rams, ewes and lambs were compared using 2-sample *t*-tests with two-tailed distributions and alpha=0.05. Differences in antibody responses against *M. ovipneumoniae* between rams, ewes and lambs were compared using Mann–Whitney test. Titers of lkt-nAb in study 3 were compared between control and treatment group ewes using two-way repeated measures analysis of variance (ANOVA), with treatment the main effect and time a within effect factor. Multiple comparisons were made between different time points for pre- and post-treatment using Sidak's multiple comparison tests. Titers of lkt-nAb pre- and post-treatment in treatment group ewes were compared using Friedman test for two-way repeated measures analysis of variance (ANOVA). Multiple comparisons were made between different time-points using Dunn's multiple comparison tests. Results were considered significant at *P*≤0.05. All analyses were carried out in Minitab 17^®^ Statistical Software (2007), (State College, PA; www.minitab.com), and GraphPad Prism^®^ version 6.00 for Windows (GraphPad Software, La Jolla, CA; www.graphpad.com).
